# "I really should've gone to the doctor": older adults and family caregivers describe their experiences with community-acquired pneumonia

**DOI:** 10.1186/1471-2296-7-30

**Published:** 2006-05-05

**Authors:** Caralyn Kelly, Paul Krueger, Lynne Lohfeld, Mark Loeb, H Gayle Edward

**Affiliations:** 1 Department of Clinical Epidemiology and Biostatistics, McMaster University, Hamilton, Canada; 2 St. Joseph's Health System Research Network, Father Sean O'Sullivan Research Centre, Hamilton, Canada; 3 Department of Pathology and Molecular Medicine, McMaster University, Hamilton, Canada; 4 Department of Family Relations and Applied Nutrition, University of Guelph, Guelph, Canada

## Abstract

**Background:**

Responding to acute illness symptoms can often be challenging for older adults. The primary objective of this study was to describe how community-dwelling older adults and their family members responded to symptoms of community-acquired pneumonia (CAP).

**Methods:**

A qualitative study that used face-to-face semi-structured interviews to collect data from a purposeful sample of seniors aged 60+ and their family members living in a mid-sized Canadian city. Data analysis began with descriptive and interpretive coding, then advanced as the research team repeatedly compared emerging thematic categories to the raw data. Searches for disconfirming evidence and member checking through focus groups provided additional data and helped ensure rigour.

**Results:**

Community-acquired pneumonia symptoms varied greatly among older adults, making decisions to seek care difficult for them and their family members. Both groups took varying amounts of time as they attempted to sort out what was wrong and then determine how best to respond. Even after they concluded something was wrong, older adults with confirmed pneumonia continued to wait for days, to over a week, before seeking medical care. Participants provided diverse reasons for this delay, including fear, social obligations (work, family, leisure), and accessibility barriers (time, place, systemic). Several older adults and family members regretted their delays in seeking help.

**Conclusion:**

Treatment-seeking delay is a variable, multi-phased decision-making process that incorporates symptom assessment plus psychosocial and situational factors. Public health and health care professionals need to educate older adults about the potential causes and consequences of unnecessary waits. Such efforts may reduce the severity of community-acquired pneumonia upon presentation at clinics and hospitals, and that, in turn, could potentially improve health outcomes.

## Background

Considerable literature exists on how seniors experience and respond to illness, including how they make decisions to use health services. Researchers have learned that many older people find it difficult to determine the importance of their symptoms, and that this "figuring-out" process is potentially time-consuming, [1,2] particularly for people already coping with chronic illness [3,4]. People's decisions to seek medical attention are also influenced by subjective assessments of their overall health and symptom severity [5,6]. Contextual factors such as location, time, and who else is involved also influence treatment-seeking decisions [3,7].

Because people do not always seek treatment promptly when severe or unusual symptoms appear, it is important that the factors influencing health behaviours are more clearly understood. Insights into illness experience and health service utilization decisions have assisted researchers studying cardiac patients, [8-10] but have yet to be incorporated into pneumonia studies.

Community-acquired pneumonia (CAP) is a serious public health concern for older Canadians who have both a higher incidence rate for pneumonia and more serious sequelae (e.g. hospitalization or death) than younger adults [11-13]. As people age, clinical signs and symptoms of pneumonia become increasingly variable. Fever, pain and cough may be minimal or absent in this population [14-17]. For health care professionals, this absence or non-specificity of symptoms and signs can make it difficult to accurately diagnose pneumonia on a clinical basis [15]. It also has implications for older adults and their families, because it may make it harder for them to recognize there is a serious health problem to be addressed, potentially causing delays in seeking care.

Anecdotally, it has long been suspected that individuals who experience vague symptoms might delay seeking treatment [15] which, in turn, can result in increased disease severity at presentation to a health care provider [18]. Knowledge about how community-dwelling older patients and their families experience pneumonia, and how they make decisions about seeking care is incomplete, [12] pointing to the need for more research on this topic.

In this paper, we report on a qualitative study that was part of a larger mixed-methods study to assess the impact of community-acquired pneumonia on older adults and their family caregivers. A key feature of the larger quantitative component was a sampling protocol that targeted an entire community so that data could be collected from both hospitalized and non-hospitalized community-dwelling older adults. The qualitative component was designed to collect rich and detailed descriptions of pneumonia experiences from a small, purposely selected sample of older adults and their family caregivers using both in-depth interviews and focus groups.

## Methods

This study was conducted in Brant County, Ontario, which includes both the city of Brantford and the amalgamated County of Brant. The population of Brant County in 2001 was 118,485 people, with 14% of the population aged 65 years and older [19]. There were two community hospitals, eight radiology centres, and approximately 80 family physicians at the time of the study. Brant County was selected for this community-wide study because of its moderate size and population demographics: it is a predominantly English speaking community, with 86% of the population reporting English as a first language [19].

An attempt was made to recruit all English-speaking community-dwelling older adults (60 years of age or older) sent for chest x-rays to confirm or rule out pneumonia at all eight of the x-ray facilities. Study participants completed quantitative telephone interviews approximately four weeks post chest x-ray. Participant recruitment for the qualitative component was then made after the telephone interview was complete. Eligibility criteria for the qualitative component included speaking English, having a radiologically confirmed diagnosis of pneumonia, and being information rich (having the ability to articulate one's experience with clarity and abundant detail). Participating older adults then identified the family member they felt fulfilled the role of primary informal caregiver. The family caregiver also had to speak English and be capable of sharing rich details on the topic under study. Ethics approval for this study was obtained from both McMaster University and the Brant Community Healthcare System.

Semi-structured interview guides for the older adults and family caregivers elicited information about participants' understanding of pneumonia (e.g., its causes, consequences, and best means for treatment), and included questions about treatment seeking and preferences for different health care providers in the community. For the older adult interview, respondents were also asked to reconstruct the actual illness episode using a calendar, and then rank symptoms according to perceived severity. Family caregivers answered specific questions about their helping role. Questions were added during the study to clarify emerging themes gathered during earlier interviews [20].

A purposeful sampling technique was used to recruit participants who were both "information rich" and could describe a variety of pneumonia experiences on the basis of symptom severity and site of treatment and convalescence. We assessed information richness during the telephone surveys, and invited participation for the in-person, in-depth interviews from those individuals who spontaneously demonstrated their effective communication skills and willingness to speak candidly about themselves. Information about each individual's pneumonia experience was also gathered during the course of the quantitative survey. We did encounter a recruitment challenge with this process, namely, a reluctance to volunteer for the in-person qualitative interview after completing the comprehensive quantitative telephone survey. According to several potential interviewees, they felt they had nothing more to say about their pneumonia. We believe that interview fatigue contributed to this reluctance to volunteer for the qualitative interview. In response to this barrier, we changed our recruitment protocol, with the permission of the appropriate ethics review board, to ending the telephone survey with a request for permission to make a follow-up phone call. With this revised strategy, we successfully recruited our final four individual interviewees plus additional participants for the validating focus groups.

Between April and December 2003, we recruited and interviewed seven older adult-family caregiver dyads (pneumonia sufferer plus his or her family caregiver) and four older adults without family caregivers (one of whom had no one to name, three of whom did not want a family member involved). Participants gave written consent for audio recording and transcription of interviews before answering questions posed by two members of the research team who were trained interviewers (GE and CK).

Four focus groups with 11 older adults and six caregivers were conducted in January 2004, two with older adults and two with caregivers. Nine of these participants (five older adults and four caregivers) had previously participated in an individual interview.

In addition to recording each interview, interviewers kept field notes documenting their perceptions about the session and documenting nonverbal data. Each recording was typed verbatim (transcribed). The interviewers then compared verbatim transcripts with the audio recordings to ensure accuracy of the transcripts and to integrate field note data into the account.

As is recommended for qualitative studies, data analysis and collection proceeded simultaneously. The interviewers independently coded the transcripts line by line, comparing codes and reaching consensus about them in weekly meetings that incorporated regular feedback from the study's principal investigator. Descriptive and interpretive codes used in this process were logged to facilitate frequent comparisons of themes within and across transcripts, and to guide a focused literature search. As new findings emerged, questions were added to the interview guides in order to clarify findings and seek additional information. An unanticipated theme, "delay in treatment seeking," emerged as an issue for many participants and so was further pursued in interviews and an expanded literature review. Using an editing approach, the team conducted a targeted exploration and diagramming of the relationships between the emergent central theme and the larger context identified in the literature [21]. As a final step, the team conducted four focus groups, two with older adults and two with family caregivers, to verify interpretations.

By the fifth participant interview, redundancy on the emergent theme of treatment seeking delay emerged. The eleventh participant provided the disconfirming case enabling us to explore the constraints or limits on delaying behaviour [22,23]. Having achieved redundancy on the theme of delay, we presented this finding to focus groups (two with older adults and two with family caregivers) for member-checking, to see whether the participants recognized the findings as being consistent with their own experiences [24]. Additionally, the focus groups provided further opportunities to seek both confirming and disconfirming evidence.

In addition to weekly team meetings about data analysis and interpretation (researcher triangulation), and maintenance of an audit trail of decisions, rigour was ensured by conducting confirmatory focus groups and by having interviewees review a written summary of our findings. Each participant validated the findings by providing us with feedback on forms that solicited both structured and open-ended replies. Situating the findings in the literature was the next crucial step because delay was an unanticipated theme that emerged from the data. In qualitative research, generalizability (transferability) is enhanced through theory, not through the comparability of the sample to the population [25].

## Results

The 11 older adults we individually interviewed ranged in age from 61 to 81 years of age (average age 71 years). Six were male. Two of them had received treatment for pneumonia in a walk-in or urgent care clinic, three from their family physician, and six in a hospital emergency room (five participants were subsequently hospitalized for between four to 11 days). Three of the people seeking care at the emergency room had sought previous treatment from a physician (see Table 1). Six of the seven members in the first older adult focus group were women, as was one of the four members in the second group.

**Table 1:** Interview participant characteristics

ID	Rating of CAP severity*	Admitted to hospital	Age	Sex	Marital status	Education level^†^	Income^‡^
P1^§^	severe	Y	72	F	married	5	5
P2	moderate	N	61	M	married	4	6
P3	severe	N	79	F	widowed	4	4
P4	very severe	Y	73	M	married	8	6
P5	moderate	N	64	F	married	7	7
P6	severe	Y	81	M	married	4	4
P7	very severe	N	78	M	common law	4	refused
P8	moderate	N	70	F	widowed	7	3
P9	moderate	N	78	F	widowed	4	4
P10	severe	Y	65	M	married	4	5
P11	very severe	Y	65	M	married	6	4

* Self assessed rating using a 5-point scale from very mild to very severe (i.e. 1 = very mild; 2 = mild; 3 = moderate; 4 = severe; 5 = very severe)

^† ^Self reported: (4 = some high school; 5 = complete high school; 6 = some community college; 7 = completed community college; 8 = some technical school)

^‡ ^Total family income in the last year before deductions: (3 = 10–19,999; 4 = 20–39,999; 5 = 40–59,999; 6 = 60–79,999; 7 = over 80,000)

^§ ^We identified our respondents by type and number (e.g. P1 refers to the first older adult, or participant, with recent experience with pneumonia that we interviewed)

Additional information about older adults and their experiences with pneumonia was collected during individual and group interviews with family caregivers. Six of the seven family caregivers were spouses (one man and five women) and the seventh was an adult daughter. Two of the four members in the first caregiver focus group were women; both members of the second group were women. With the exception of one adult daughter, all caregivers in the focus groups were spouses.

In each interview with older adults, we were told about delays in seeking professional medical care. The most striking comments were from participants who strongly regretted waiting and felt they "should have" sought medical attention sooner. Other participants simply explained when different symptoms developed and identified factors that led them to wait. The retrospective illness episodes constructed during the interviews revealed delays ranging from a few days to over a week. As well, advice for other older adults with pneumonia that was elicited at the end of each interview strongly urged people to promptly seek care, especially if symptoms are severe or persistent. Similar accounts of delaying behaviour were also reported in the focus groups.

Several of the patients who reported experiencing the most serious pneumonia episodes indicated that they attributed the illness severity, at least in part, to their delay. A typical statement was, "Well, I never went to the hospital before. Just antibiotics. You know, you get sick, you go to the doctor. But this time it went on too long" [P4].

There were exceptions to this pattern. One interviewee described how the severe and persistent chest pain he felt over several hours led him to fear that he was experiencing heart troubles, so he called '911'. Similarly, a focus group participant described how her children interpreted her symptoms as stemming from heart trouble, thus expediting their decision to seek care for her at a local ER. Prompt decision-making was also described by another group participant whose history with respiratory conditions led her take immediate action when familiar symptoms appeared. Although these accounts do demonstrate limits for delay, they did not absolutely negate its existence, only its reduced duration from days or weeks to hours.

Confirmation of and concern about delaying behaviour was often described by family caregivers. In both the individual and group interviews, family caregivers recalled help-seeking delays with regret and even anger. They told us they wish they had "insisted" or "forced" their family member to seek care sooner.

Data revealed three different phases of delay. Immediately following symptom onset, respondents described appraisal challenges (Table 2). Generally, people who delayed seeking care either did not believe they had a serious condition or they mistakenly attributed the symptoms to other, pre-existing health problems. In the second phase, older adults recognized they were sick, but were not yet ready to conclude that professional medical care was required (Table 3). In this phase, people initiated self-care (e.g., taking home remedies and/or over-the-counter medications, changes to diet and behaviour) and monitored how their symptoms responded. This process lasted from a few days to over a week. During the final phase, the older adults we spoke with recognized that professional medical help was required, but other circumstances or issues prevented them from seeking help promptly (Table 4). A wide array of explanations accounted for delays in this phase: inconvenient time (e.g., evenings, weekends), inconvenient locations (e.g., away on holiday), access barriers (e.g., inability to obtain a timely medical appointment), social or work obligations, fear of the consequences of seeking help (e.g., unpleasant treatment procedures, a diagnosis of an incurable condition), and not wanting to bother other people (e.g., asking for a ride to the emergency room).

**Table 2:** Is something wrong?*

"I wasn't convinced that it was something serious as pneumonia" [P9]
"I thought it was just a cold, and I thought, 'Well, this will go away" [P3]
"I always ... figured if you have a cold and don't look after it, it will turn into pneumonia. ... That wasn't the case with me. I didn't have a sign of a cold." [P8]
"He was coughin' and coughin' a lot. [But] he coughed a lot anyways.... I didn't realize it had affected his breathing 'cause he had a breathing problem anyways." [CG7]

* Following accepted practice, we have edited statements to increase clarity without altering meaning. Ellipses (...) indicate where words have been removed. Words placed in square brackets [ ], indicate where words have been added to respondents' statements. The abbreviation CG refers to caregiver, FGP to participant focus group.

**Table 3:** Do I need medical help?

"I noticed I was getting sick .., I thought maybe it'd go away but it didn't." [P3]
"I put up with it for about eight or 10 days before I went to the doctor 'cause I thought it was just something that would go away. But, [laughs] it didn't go away, it just kept on getting' worse." [P7]
"I was pretty sick this time. My temperature was 104 for about three nights in a row. I kept thinking I was going to just get rid of it myself because that has happened before." [P1]
"People kept telling me I had pneumonia. They figured I had pneumonia, and I thought, 'Well, I haven't got pneumonia 'cause I had the pneumonia shot a year ago' [and that should have protected me], not knowing that there's different types of pneumonia." [P10]

**Table 4:** Do I need medical help right now?

"I will not go to a hospital because of my fear of the emergency room.... They gave me drugs I was allergic to.... that can be scary." [FGP1]
"I had pain with [breathing] right in the left side. That's when I thought, 'Oh, God! Don't tell me this lung is gonna go [too!].' . . . . [They] chop [ped] the right [lung] out. [Will they take the] left out now?'... I thought, 'If I go to the doctor, he's going to tell me that I got another thing in the lung, and I'm going to have another operation, and oh, my God!" [P3]
"I probably would've gone to my own doctor [right away] but going to [a doctor elsewhere, like while on vacation] – you have to go to a clinic and you have to wait so long while [at home], the doctor I go to is very, very good." [P11]
"You put it off because you think, 'Oh, I just can't take time off today, 'cause I've got this, this, and this to do.' ...It's easy to do." [FGP1]
"One reason I didn't go [to the emergency room] Sunday night – I wasn't gonna go and sit down there and [wait] four or five hours to be looked at." [P10]
"And when I called Dr. [X] he didn't have no openings ... so I had to wait. ... He couldn't see me. He was booked too far ahead." [P7]

Three participants had sought treatment from more than one physician. Each of them described delays that they attributed to the outcome of their initial visit: an initial diagnosis of a non-serious illness that led them to downplay their symptoms even as they worsened [P1, P4], or receipt of medication offered reassurance even when symptoms failed to improve [P3]. Ultimately, all respondents sought care when either persistent or severe symptoms overrode their previous objections to care-seeking, or a family member took the initiative and either persuaded or forced the older adult to seek care.

During the focus groups, participants brainstormed to generate ideas on how to prevent care-seeking delays in older adults. Their ideas centred on education directed towards older adults like themselves. Specifically, they felt that older adults need information on responding to ambiguous symptoms, the potentially serious or even fatal consequences of CAP, the limited effectiveness of some antibiotics, and some of the more typical obstacles to care-seeking (e.g. difficulties with transportation and unwillingness to bother physicians or one's family).

The key message from the focus groups was that health care providers need to better inform seniors about what pneumonia is, what the sequelae or consequences can be, and what the criteria are for an appropriate response. Interestingly, the idea of education also arose in the interviews when older adults were asked for their advice. They tended to take responsibility for delays, rather than thinking about what health care providers might do differently. A typical comment was, "But it was me that should've gone sooner; I feel that I'm the one that could stand improving. By the time I got to them [the health care providers], they did all they could do" [P11].

## Discussion

Our results illustrate that treatment-seeking delay was a central feature of the pneumonia experience for community-dwelling older adults in a mid-sized community in South Central Ontario. The qualitative data we gathered provided details that highlighted the variability and complexity of help-seeking delays. Individually, the participants identified different barriers to seeking care and varying thresholds where a perceived need for care finally overrode the reasons for waiting. Respondents also expressed varying degrees of regret for their delays. Some profoundly regretted their behaviour, but others told us that if future illnesses occurred in similar circumstances, they would likely delay again.

Although the range of responses emphasized the variability of care-seeking delays, the consistency of the overall experience was significant. Moreover, our findings are consistent with the three-stage model of delay proposed by Safer *et al *[1]. In the first stage (appraisal delay), people assess whether sensations indicate something is wrong. In the second stage (illness delay), individuals evaluate whether the health problems will be improved by professional care. In the final stage (utilization delay), they decide whether the care is worth the costs and the efforts to overcome barriers.

The key feature of this model is its conceptual identification of different components of delay, and its corresponding emphasis that researchers and clinicians not view delay as an undifferentiated totality. As well, considering delay as part of a decision-making process highlights how different factors (e.g., sensory, emotional, situational, etc.) may be important at different stages, and thus require different types of interventions to respond to them. For example, the appraisal delay experienced by older adults who found their symptoms difficult to interpret (e.g., malaise but no cough) can be expected with pneumonia because of the disease's variable clinical presentation in older adults. Thus, future researchers may want to explore the CAP-specific threshold for appropriate and inappropriate delays in treatment seeking. The examples of emotional and situational factors comprising utilization delays suggest a different focus for intervention. Patients need to be guided towards more rational decision-making by educating them about the potential pitfalls that impede reasoning and lead to decisions to extend the wait before seeking appropriate professional care [3]. Importantly, research has shown that sensory, perceptual and situational factors affecting treatment decisions are modifiable through educational interventions [1,26].

Anecdotal accounts in the pneumonia literature indicate that the longest delays in obtaining treatment occur in the pre-hospital/clinic phase [18], a finding that is also well established in the literature on heart attacks [8-10,27]. Because earlier administration of antibacterial therapies results in improved clinical outcomes, [15,28,29] the need for patient and health care provider education is important if we are to reduce the amount of time between symptom onset and treatment.

There are several limitations to this study. Although delay was the pivotal theme that emerged from our interviews (and was corroborated in the focus groups), the methodological design meant that only people who actually sought treatment resulting in an x-ray were eligible to participate. Although this design ensured that we were studying a population that shared an experience (i.e., CAP), we did not capture people who sought alternative care, had milder cases of pneumonia and recovered without professional treatment, or were treated by a physician who did not order an x-ray. The findings from these populations may be different.

Our findings may also be limited by sample size. Although qualitative studies can generate credible findings from as few as six respondents, a larger sample size will enable researchers to reach saturation on multiple themes or subthemes. In this study, we reached saturation on the primary theme, care-seeking delay, and confirmed its validity in both focus groups and the literature. However, additional participants would have permitted exploration of individual-level factors. Due to the recruiting challenge described earlier, we did not have the opportunity to expand our study further. Future researchers may benefit from our experience, particularly when designing qualitative studies embedded within a larger quantitative research program. Qualitative research is strengthened by the enthusiastic participation of respondents who genuinely want to share their experiences. Thus researchers need to be aware of recruiting protocols that may hinder recruitment. Instead of asking for volunteers immediately after people complete a survey, we recommend making requests for additional participation in a study after a rest period of a few days.

This study provides an example of how qualitative research can identify underlying reasons for a behaviour, but not its prevalence. To answer that type of question, well-designed quantitative surveys with appropriate numbers of older adults randomly selected from the community would be needed. A quantitative investigation would elicit data on the characteristics of individuals more likely to delay seeking help. These include modifiable psychosocial and contextual factors, along with the demographic characteristics that can trigger healthcare providers to be more vigilant in promoting prompt healthcare for this group. The collection of longitudinal data may be key because many participants in our study told us, "I delayed *this time *because ...", thus indicating the relative importance of contexts and environments compared to more fixed individual variables.

Although retrospective studies like this one may be limited by the reduced accuracy of the data based on respondents' recall, we took measures to offset this. First, we scheduled interviews within four to eight weeks of a chest x-ray to allow adequate time for recovery, yet still be recent enough for respondents to accurately report their experiences. We also encouraged respondents to use memory aids, such as calendars and prescription receipts. A prospectively designed study employing health diaries may elicit additional data, although the quality of diarized data collected over a protracted period of time without prompts by researchers may be questionable.

The context in which our data were collected should also be considered when interpreting the findings. Canada has a publicly funded system. The guiding philosophy is that all Canadians should have equal access to health care, regardless of their ability to pay. Nevertheless, our respondents identified access barriers that were factors (e.g., long waits in hospital ERs, walk-in clinics and urgent care facilities, as well as difficulties getting an appointment with one's family doctor) that contributed to their decision to delay seeking care. In addition, the Canadian media's frequent reporting of Canada's shortage of family physician's, health care funding shortfalls, and overcrowded ER's could also have led some people to delay seeking care until they could no longer comfortably function in their daily lives.

## Conclusion

Delay in treatment seeking for symptoms of CAP was an emergent finding in this qualitative study designed to describe the pneumonia experiences of community-dwelling older adults and their family caregivers. Respondents spontaneously identified help-seeking delay as an important issue. In-depth exploration of pneumonia episodes revealed that delay was part of a variable and multi-phased decision-making process. Symptoms were important, ultimately leading people to seek care, but were variously interpreted based on psychosocial and situational factors. Educational efforts focusing on the causes and consequences of unnecessary waits should be developed and directed towards older adults. With the literature showing that early treatment of CAP can lead to improved health outcomes, more efforts need to be directed to develop and test initiatives designed to reduce help-seeking delays among older adults in the community.

## Competing interests

The author(s) declare that they have no competing interests.

## Authors' contributions

CK had a role in the design of the qualitative study, collection of data, data management, analysis of results and was the lead writer of this manuscript.

PK had a major role in the conception and design of the study, supervised all aspects of the study's implementation, contributed to the writing of the manuscript and provided editorial comments.

LL had a major role in the conception and design of the qualitative study, contributed to the writing of the manuscript and provided editorial comments.

ML contributed to the conception of the study, critically reviewed the manuscript and provided editorial comments.

GE had a role in the design of the qualitative study, collection of data, data management, analysis of results and the critical review of the manuscript.

## Pre-publication history

The pre-publication history for this paper can be accessed here:


